# IRIXS: a resonant inelastic X-ray scattering instrument dedicated to X-rays in the intermediate energy range

**DOI:** 10.1107/S1600577519017119

**Published:** 2020-02-26

**Authors:** Hlynur Gretarsson, Didem Ketenoglu, Manuel Harder, Simon Mayer, Frank-Uwe Dill, Manfred Spiwek, Horst Schulte-Schrepping, Markus Tischer, Hans-Christian Wille, Bernhard Keimer, Hasan Yavaş

**Affiliations:** a Max-Planck-Institut für Festkörperforschung, Heisenbergstrasse 1, D-70569 Stuttgart, Germany; b Deutsches Elektronen-Synchrotron DESY, Notkestrasse 85, D-22607 Hamburg, Germany; c Department of Engineering Physics, Faculty of Engineering, Ankara University, Ankara 06100, Turkey; d SLAC National Accelerator Laboratory, 2757 Sand Hill Road, Menlo Park, CA 94025, USA

**Keywords:** IRIXS beamline, Petra III, DESY, RIXS, beamlines

## Abstract

A new resonant inelastic X-ray scattering (RIXS) instrument has been constructed at beamline P01 of the PETRA III synchrotron. This instrument has been named IRIXS (intermediate X-ray energy RIXS) and is dedicated to X-rays in the tender-energy regime (2.5–3.5 keV). The range covers the *L*_2,3_ absorption edges of many of the 4*d* elements (Mo, Tc, Ru, Rh, Pd and Ag), offering a unique opportunity to study their low-energy magnetic and charge excitations. The IRIXS instrument is currently operating at the Ru *L*_3_-edge (2840 eV) but can be extended to the other 4*d* elements using the existing concept. The incoming photons are monochromated with a four-bounce Si(111) monochromator, while the energy analysis of the outgoing photons is performed by a diced spherical crystal analyzer featuring (102) lattice planes of quartz (SiO_2_). A total resolution of 100 meV (full width at half-maximum) has been achieved at the Ru *L*_3_-edge, a number that is in excellent agreement with ray-tracing simulations.

## Abstract 2

A resonant inelastic X-ray scattering instrument operating in the intermediate X-ray range is described in detail. The instrument is operated at beamline P01 of the PETRA III synchrotron in Hamburg, Germany.

## 1. Introduction   

Over the last two decades, resonant inelastic X-ray scattering (RIXS) has evolved from a technique primarily used to probe charge-transfer or *d–d*-excitations in the 3*d* transition metals (TMs) (Grenier *et al.*, 2005 ▸; Huotari *et al.*, 2008 ▸; Abbamonte *et al.*, 1999 ▸; Kim *et al.*, 2002 ▸) to a powerful tool for mapping out entire magnon dispersion relations in correlated material systems (Ament *et al.*, 2011 ▸; Dean, 2015 ▸). The discovery of spin-flip excitations being allowed within the *L*_3_-edge RIXS process was a major boost for this achievement. After the first results in the soft X-ray regime (Cu *L*_3_-edge, 2*p*_3/2_ → 3*d*, ∼940 eV) (Braicovich *et al.*, 2010 ▸), magnon dispersions were measured utilizing the Ir *L*_3_-edge (∼11 keV) (Kim *et al.*, 2012 ▸). Coincidentally, there have been major advancements on the instrumentation front, pushing energy resolution to levels that allow resolving low-energy excitations. Improvements in gratings (lower slope error) for soft X-rays as well as the use of higher-order crystal reflections (closer to backscattering) with position-sensitive detectors for hard X-rays make sub-100 meV energy resolution a routine for many of the 3*d* and 5*d* TM *L*_3_-edges (Brookes *et al.*, 2018*b* ▸; Strocov *et al.*, 2010 ▸; Moretti Sala *et al.*, 2018 ▸; Shvyd’ko *et al.*, 2012 ▸; Ishii *et al.*, 2013 ▸).

The ability of RIXS to probe collective modes, such as magnetic excitations typically found in the 10 meV or 100 meV range, has been among the main drivers for the construction of new RIXS beamlines. However, the strict optical requirements for these instruments have thus far limited RIXS studies primarily to the 3*d* TMs, such as superconducting cuprates (∼930 eV) (Tacon *et al.*, 2011 ▸), and 5*d* TMs, such as strongly spin–orbit-coupled iridates (∼11.215 keV) (Gretarsson *et al.*, 2016 ▸). *L*_3_-edges of the 4*d* TMs, falling in the intermediate (often referred to as tender) energy range, had been precluded simply due to the lack of suitable optical schemes.

Although progress has been made for X-ray scattering and spectroscopy techniques requiring low/moderate energy resolution at the *L*_3_-edges of the 4*d* TMs [*e.g.* XAS (Barla *et al.*, 2016 ▸), XES (Brookes *et al.*, 2018*a* ▸), REXS (Strempfer *et al.*, 2013 ▸; Collins *et al.*, 2010 ▸)], very little has been achieved when it comes to higher energy resolution (<100 meV). For a realistic grating (1200 lines mm^−1^), a resolving power of <20000 can be achieved (Viefhaus *et al.*, 2013 ▸), which corresponds to >140 meV at the Ru *L*_3_-edge (2.840 keV), but the reflectivity is expected to be only a few percent. For crystal reflections, on the other hand, the relatively low energy of the tender X-rays severely limits the available Bragg reflections. For instance, (111) is the only Bragg reflection available in silicon at the Ru *L*_3_-edge of 2840 eV with an intrinsic bandwidth of ∼370 meV. Compared with cubic silicon, lower-symmetry crystals such as sapphire and quartz offer orders of magnitude more unique Bragg reflections to choose from, which have been demonstrated to have the required quality for RIXS applications (Yavaş *et al.*, 2007 ▸, 2017 ▸; Gog *et al.*, 2013 ▸; Ketenoglu *et al.*, 2015 ▸; Said *et al.*, 2018 ▸).

In this article, we will describe the design of our IRIXS instrument (intermediate X-ray energy RIXS) and provide experimental data demonstrating its performance. The newly developed instrument, located at beamline P01 of the PETRA III synchrotron in Hamburg, has already proven to be capable of measuring the RIXS spectra of Ru-based compounds using the large resonance at the Ru *L*_3_-edge (2840 eV) (Suzuki *et al.*, 2019 ▸; Gretarsson *et al.*, 2019 ▸). The beamline design is primarily based on hard X-ray optics, using Bragg reflections from near-perfect single crystals rather than gratings that are typically used at soft X-ray instruments. The novel optical design in combination with a quartz-based analyzer gives a total resolution of 100 meV (full width at half-maximum).

## 2. Beamline overview   

**Figure 1:**
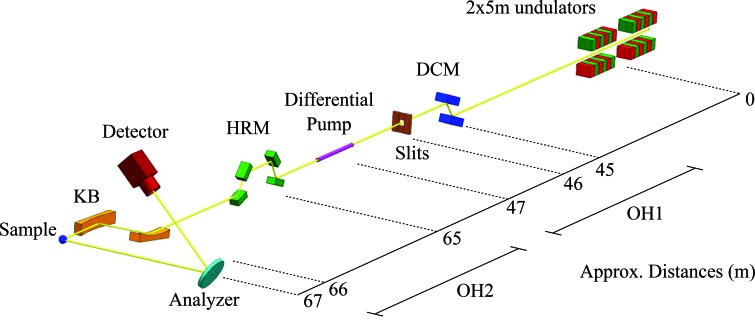
Layout of the IRIXS instrument at P01 showing the position of the beamline components with respect to the center of the undulators. The beam propagates from right to left, going through multiple elements (see text for details) before hitting the sample and is subsequently analyzed by the spectrometer.

Fig. 1 ▸ shows the schematic layout of beamline P01 with emphasis given to components that are relevant to IRIXS [for more general details on P01, see the work by Wille *et al.* (2010 ▸)]. P01 was originally constructed as a hard X-ray beamline that specializes in nuclear resonant scattering (NRS) and inelastic X-ray scattering (IXS), with a minimum photon energy of 6 keV. In 2017, two new undulators extended the photon range down to 2.5 keV. At these low energies, air absorption and attenuation by windows become significant, which requires that IRIXS is fully in vacuum and operating without any windows. To facilitate this, we installed the instrument as close to the machine vacuum as possible, converting the old secondary optical hutch (OH2) to a new experimental hutch. A differential pump, located immediately upstream of the IRIXS instrument, allows operation at a vacuum level of 10^−6^ mbar, which is inferior to the accelerator level. The double-crystal monochromator (DCM), which consists of a pseudo channel-cut Si(111) with liquid-nitrogen cooling, is located upstream of the IRIXS instrument and the differential pump. The crystal surfaces of the DCM are cut with an asymmetry angle of α = 32° in order to increase the footprint of the high-power undulator beam on the first crystal, consequently reducing the ensuing heat-load density. Since the asymmetry is opposite on the second crystal, both the beam size and the angular divergence stay intact after the DCM. However, this asymmetric geometry results in a bandwidth around 600 meV at 2840 eV, about twice as large as that of a symmetric Si(111) reflection. After the DCM, a four-blade slit system defines the beam in real space before the high-resolution monochromator (HRM), which is located in the hutch that hosts the IRIXS instrument. A four-bounce HRM (4B-HRM) further defines the photon energy with a bandwidth around 60 meV. The monochromatic beam is focused on the sample with a Kirkpatrick–Baez (KB) mirror system before being analyzed (both in momentum and energy) by the spectrometer. From our calculations we have estimated the photon flux at 2.840 eV to be 7 × 10^13^ photons s^−1^ (after the DCM) and 1 × 10^12^ photons s^−1^ (after the HRM). With such a flux a typical measurement time does not exceed 1 h. This, however, depends on the energy range and statistics required. In the following sections we will describe in detail the design and performance of the IRIXS components.

## 3. High-resolution monochromator   

**Figure 2:**
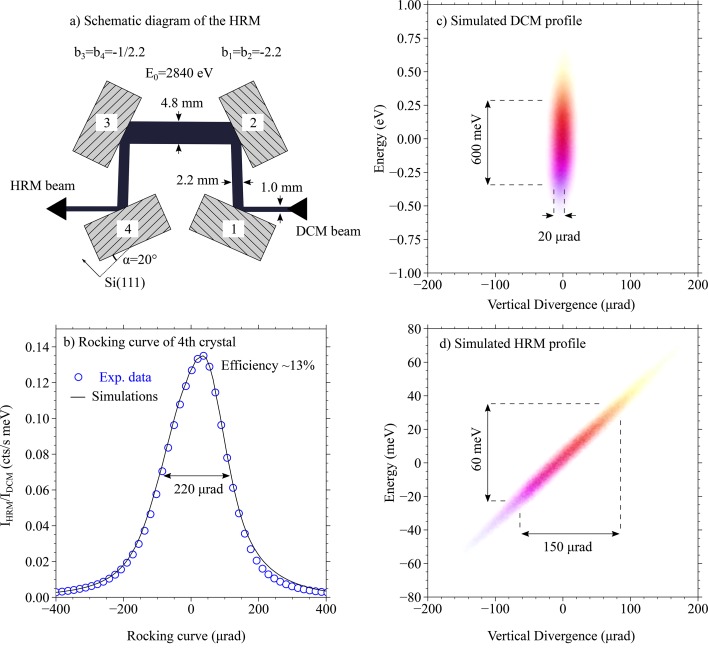
(*a*) Schematic diagram of the HRM: it consists of four Si(111) crystals cut asymmetrically with an angle α = 20°. Reflections 1 and 2 collimate the beam while 3 and 4 select the energy. (*b*) Comparison between the experimental rocking curve of crystal number 4 and the simulations. Efficiency of ∼13% is achieved. (*c*) Calculated phase space of the photons entering the HRM showing a well collimated beam with a large energy bandwidth. (*d*) As (*c*) but for the photons exiting the HRM. Here the bandwidth is reduced by a factor of ten but the divergence increases.

In Fig. 2 ▸(*a*), a schematic diagram of the HRM can be seen. This novel design was originally proposed for NRS beamlines in order to provide a narrow energy bandwidth (∼1 meV) at the Fe nuclear resonance (14.41 keV) (Toellner, 2000 ▸; Yabashi *et al.*, 2001 ▸). However, the design can be generalized to arbitrary energies, such as around the Ru *L*_3_-edge. Our monochromator consists of four Si(111) crystals placed in the (+ − − +) configuration (Shvyd’ko, 2004 ▸). For the HRM, silicon crystals were picked since other materials, such as quartz or sapphire, have been proven unstable due to heat load (Gog *et al.*, 2018 ▸). Each silicon crystal is cut asymmetrically with an angle α = 20° (Bragg angle θ_B_ = 44.12°), which corresponds to an asymmetry parameter *b*_1,2_ = −sin(θ_B_ + α)/sin(θ_B_ − α) = −2.2 for the first and second crystals, whereas the third and fourth have *b*_3,4_ = −1/2.2. The asymmetric parameter *b* re-normalizes the energy bandwidth of each crystal according to Δω′ = Δω × (|*b*|)^1/2^, where Δω = 370 meV is the bandwidth for a symmetric Si(111) at ω = 2840 eV (Shvyd’ko, 2004 ▸). It then follows that the first two reflections have a bandwidth of Δω′ = 560 meV, whereas for the latter two it is Δω′ = 250 meV.

The asymmetry parameter *b* can also be used to estimate the size of the reflected beam using the relation *z*′ = *z* × |*b*|, where *z* and *z*′ are the sizes of the incoming and outgoing beams, respectively, along the dispersive direction of the diffracting crystal. Since *b*_3,4_ are the reciprocals of *b*_1,2_, the vertical beam size remains the same upon exiting the HRM. Moreover, the outgoing X-ray beam direction is maintained so that the incoming and outgoing beams are co-linear.

To calculate the performance of the HRM, we used the ray-tracing software *xrt* (Klementiev & Chernikov, 2014 ▸). Calculations were simplified by using a geometrical source with a Gaussian profile centered at 2840 eV, and realistic beam size and divergence. In Fig. 2 ▸(*b*), we show the experimental data as a result of rocking the last crystal and monitoring the reflected intensity normalized to the intensity and bandwidth of the broadband beam after the DCM. The overall efficiency of the HRM is only 13% due to the low reflectivity of each Si(111) reflection, which at 2840 eV is ∼65%. The data are in good agreement with simulations, underlining the performance of the HRM as expected. In Figs. 2(*c*) and 2(*d*), we show the simulated energy and angular profiles of the broadband beam after the DCM and the high-resolution beam after the HRM, respectively. While the energy bandwidth is reduced from 600 meV to 60 meV, the divergence of the beam increases from ∼20 µrad to ∼150 µrad.

Increased angular divergence of the HRM beam is expected due to the uncompensated asymmetric crystals. In analogy to an optical prism, an asymmetric crystal introduces a dispersion rate to the reflected beam (Shvyd’ko *et al.*, 2006 ▸). This dispersion rate can be compensated when using a crystal pair (like in channel-cut crystals), but such pairs are absent in the current (+ − − +) configuration. Instead, the rate is amplified as the photons progress through the HRM, going from 0.2 µrad meV^−1^ after crystal 1 to 2.5 µrad meV^−1^ after crystal 4 as shown in Fig. 2 ▸(*d*). Due to this effect, the current HRM can reduce the bandwidth down to 60 meV, a number that would be closer to 200 meV if symmetric crystals were used. In other words, one trades between the energy bandwidth and the vertical divergence of the beam. We note that the main parameters described above (energy bandwidth and angular divergence) hold for any energies between 2.5 keV and 3.5 keV. This means that a 60 meV bandwidth is possible for other 4*d* *L*_2,3_ absorption edges.

## 4. Focusing   

An Ni-coated Kirkpatrick–Baez (KB) mirror is used to focus the beam. Each mirror has an optical length of 280 mm and is operated at a 3.5 µrad glancing angle. The vertical and horizontal mirrors have focal distances of around 1 m and 0.7 m, respectively. The KB mirror accepts a beam of 1 mm × 1 mm, which captures the full vertical profile but only two-thirds of the horizontal beam. The DCM beam can be focused down to 10 µm × 10 µm (H × V); however, the focused beam size increases to 40 µm × 150 µm (H × V) with the HRM. This drastic difference in the vertical profile is due to the increased vertical divergence introduced by the HRM. As explained by Huang *et al.* (2012 ▸), a 4B-HRM featuring uncompensated asymmetric crystals will create a virtual source at ∼0 m (see Fig. 1 ▸), whose vertical size is on the order of a millimetre instead of a micrometre, thus limiting the focusing ability. The origin of the wider horizontal profile is less clear but can originate either from a misaligned HRM, which would increase the horizontal divergence, or from a not fully optimized mirror. However, this does not affect the performance of the overall instrument.

**Figure 3:**
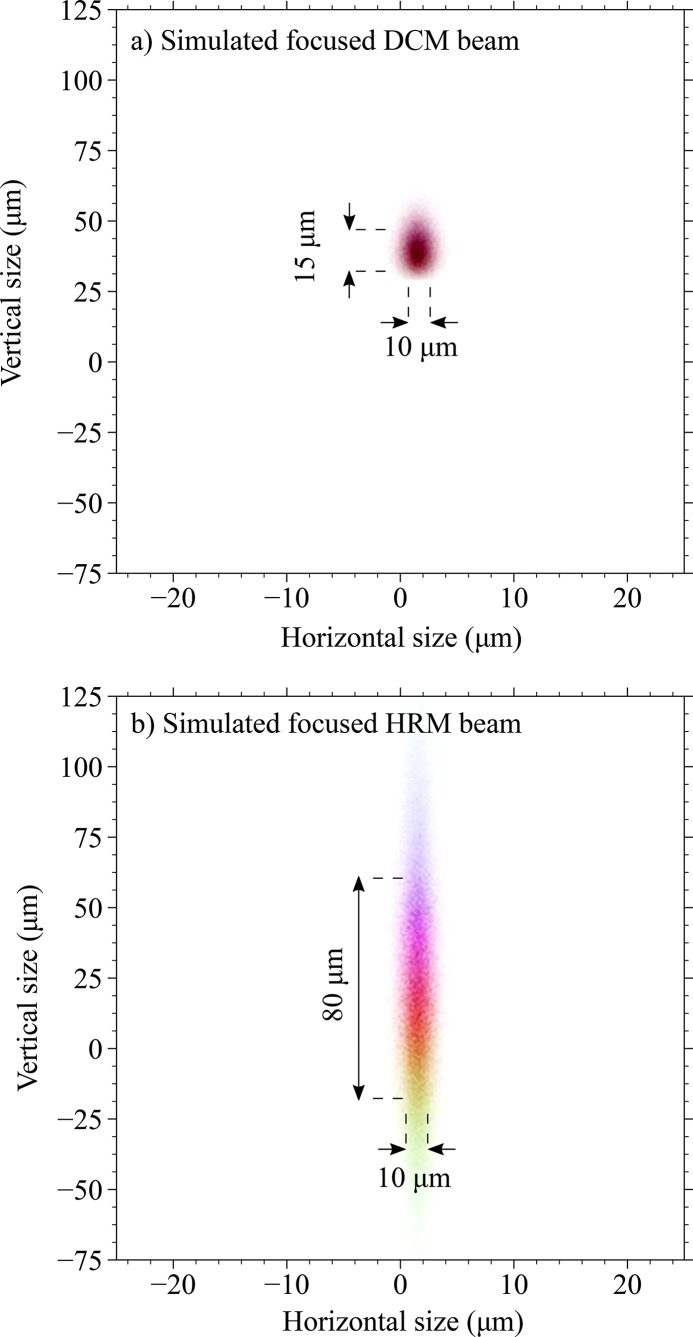
(*a*) Simulation of the DCM beam after focusing (the parameters can be seen in Fig. 1 ▸). No slope error was introduced to the mirrors but the image of the beam was recorded a few millimetres off the focal point to obtain a finite beam size. (*b*) The same configuration as in (*a*) after inserting the HRM into the beam. The vertical size of the beam increases from 15 µm to 80 µm and acquires an energy dispersion in the vertical direction.

In order to demonstrate the increased vertical beam size, we have carried out a ray-tracing simulation of the KB mirror. The images in Fig. 3 ▸ demonstrate the simulation results a few millimetres away from the focal point along the optical axis in order to mimic the finite size of the focused beam due to slope error, which is not taken into account in the simulations. The simulation estimates a substantial increase in the vertical beam size, from 15 µm to 80 µm, between the DCM beam and the HRM beam. The horizontal beam size remains the same. Additionally, the results show that, due to the vertical dispersion introduced by the HRM, different energies are projected onto different vertical positions, creating an image resembling a ‘rainbow’. However, since the beam in the vertical direction is around 150 µm, we assume that the energies remain scrambled rather than separated.

## 5. Spectrometer   

**Figure 4:**
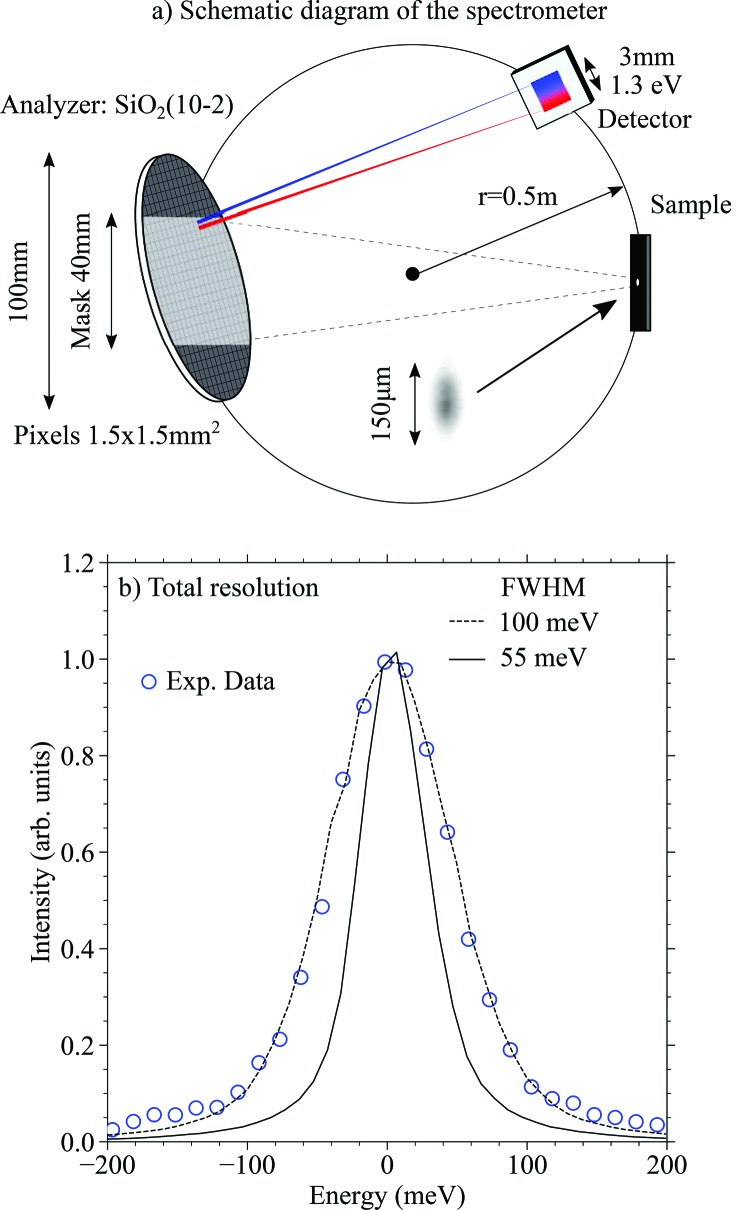
(*a*) Schematic of the spectrometer showing the position of the sample, analyzer and detector. For clarity, objects in the picture are not kept to scale. (*b*) Total energy resolution of the IRIXS instrument (blue circles) in comparison with simulations (dashed line) showing the 100 meV resolution. The intrinsic resolution of the analyzer crystal is 55 meV (solid line).

To measure both the energy and the momentum transfer of the scattered photons, the analyzer and the detector are placed on a Rowland circle as shown in Fig. 4 ▸(*a*). The spectrometer can be rotated continuously in the horizontal plane (2θ = 80° to 100°) to change both the direction and the amplitude of the momentum transfer vector.

The analyzer is based on an SiO_2_(102) wafer (θ_B_ = 73.13°), diced into segments of 1.5 mm × 1.5 mm (*l*_ana_ = 1.5 mm) with a gap of 0.2 mm. The segmented wafer is bonded on a substrate such that the end result is essentially a mosaic of flat SiO_2_ segments forming a spherical surface with a radius *R* = 1 m. For more details on the analyzer fabrication see the work by Ketenoglu *et al.* (2015 ▸). A rectangular mask (not shown) is located between the sample and the analyzer, limiting the vertical exposure of the analyzer to 40 mm. Due to the finite segment size of the diced analyzer, X-rays scattered from the sample lead to an angular variation on the flat surface, which results in a finite bandwidth of Δ*E*_f_ = 1.3 eV. This energy window, which is measured without scanning the spectrometer, can be calculated as
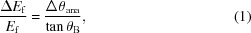
where the angular acceptance of each analyzer segment is Δθ_ana_ ≃ *l*_ana_/*R*. The energy-analyzed beam is focused on the detector, which is an Andor iKon L CCD camera (2048 × 2048) with a pixel size of 13.5 µm × 13.5 µm. The beam is energy-dispersed along the vertical direction on the position-sensitive detector, covering 3 mm of our detector with a dispersion rate of 5.8 meV pixel^−1^.

Fig. 4 ▸(*b*) shows the total experimental resolution of our spectrometer in comparison with the ray-tracing results (dashed black line). For the simulations, we used the HRM beam shown in Fig. 2 ▸(*d*) with an experimental spot size of 150 µm [uniform energy distribution, see inset in Fig. 4 ▸(*a*)]. The results agree with the measurements, giving a resolution of 100 meV. There are various factors contributing to the resolution, the largest of them being: (1) the incoming bandwidth Δ*E*_i_ = 60 meV, (2) the intrinsic resolution of the analyzer Δ*E*_a_ = 55 meV, and (3) geometrical contributions of which the Johann aberration Δ*E*_J_ and spot size Δ*E*_s_ play a dominant role.

To estimate the geometrical contribution, we performed additional ray-tracing simulations using Δ*E*_i_ = 1 meV and varied the vertical size of the spot as well as the size of the analyzer mask. The simulation results suggest Δ*E*_s_ = 60 meV and Δ*E*_J_ < 10 meV. We note that, if a full analyzer is used (no mask), Δ*E*_J_ would be >100 meV, emphasizing the importance of masking analyzers when working away from back-scattering. This drastic increase in Johann error when the edges of the analyzer are exposed simply stems from the larger mismatch between the analyzer surface and the Rowland circle (Moretti Sala *et al.*, 2018 ▸). Now, assuming normal distributions of all these errors, the total resolution can be calculated using Δ*E*_total_ = 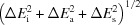 = 100 meV, which is in a good agreement with the ray-tracing results.

In Fig. 4 ▸(*b*) we show the intrinsic resolution of the analyzer crystal as a solid black line. The width of the curve is around 55 meV, suggesting the current resolution can be improved further by reducing the bandwidth and eliminating the source size error, the latter normally being <10 meV at hard X-ray beamlines (Moretti Sala *et al.*, 2018 ▸). This can be achieved by replacing the current in-line 4B-HRM with a nested 4B-HRM as pointed out by Huang *et al.* (2012 ▸).

Unlike our HRM, new analyzers are required in order to expand to other atomic resonances. For instance, moving from the Ru to the Rh *L*_3_-edge (2.840 keV to 3.004 keV) would require a different quartz reflection. At 3 keV, SiO_2_ has a (200) reflection at θ_B_ = 76.25°, close to our current operations. The calculated spectrometer performance of such a setup would be *E*_a_ = 100 meV. We note that switching analyzers for different resonances is a common practice within hard X-ray RIXS beamlines and is carried out in order to obtain the most suitable energy resolution each time.

**Figure 5:**
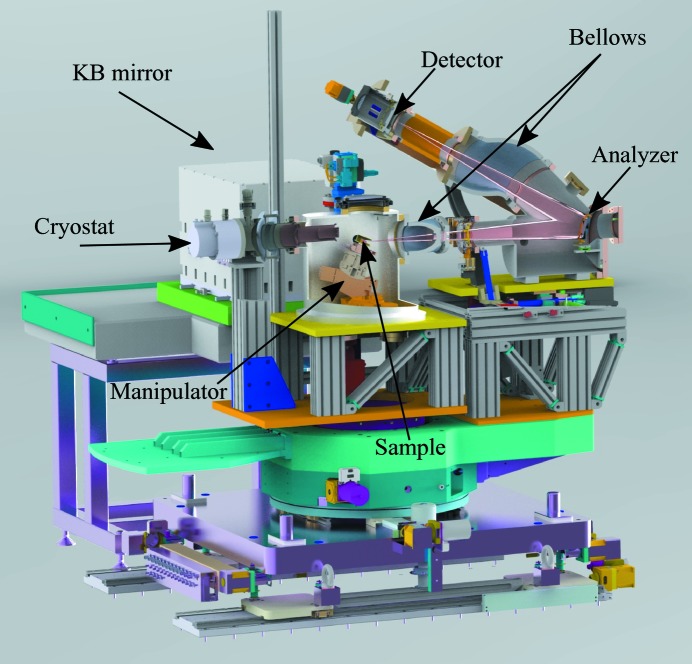
Drawing of a section of the IRIXS instrument, including the KB mirror, sample chamber and spectrometer. Sample change is carried out through a window at the top of our sample chamber. Copper braids (not shown) provide thermal contact between the cryostat and the sample holder. A set of two bellows allows us to change the angular position of the analyzer and detector.

Lastly, we show a drawing of our IRIXS instrument in Fig. 5 ▸. Incoming photons are focused on the sample using our KB mirror. The sample is mounted on top of an in-vacuum five-dimensional manipulator (θ = [0°, 90°], χ = [−5°, 35°], *x*, *y*, *z* = ±6 mm). The sample can be cooled down to *T* = 20 K using a closed-cycle He cryostat, thermally connected via copper braids (not shown). Bellows are located between the sample chamber and analyzer as well as between the analyzer and detector; this allows us to change the amplitude of the momentum transfer and scan energy loss, respectively. Sample change is performed by opening a window on top of the sample chamber. Both the venting and the subsequent pumping take approximately 1 h.

## 6. Conclusions   

Here, we have described the IRIXS instrument located at beamline P01 of the PETRA III synchrotron, DESY. The instrument is currently operating at the Ru *L*_3_-edge (2.840 keV) with the possibility to extend to other 4*d* materials. Our main components are a dispersive four-bounce monochromator, KB-optics and a Rowland spectrometer featuring a diced analyzer and a 2D detector. We have demonstrated that each component contributes equally to our resolution of 100 meV, a number that matches well with simulations. Our novel concept demonstrates that relatively high resolution can be achieved in the tender X-ray range using crystal optics. Looking further ahead, a flat-crystal analyzer in combination with collimating optics (Kim *et al.*, 2016 ▸) could provide further improvements to resolution, similar to what has been achieved at the Ir *L*_3_-edge (Kim *et al.*, 2018 ▸).
